# Orchestrated transcription of biological processes in the marine picoeukaryote Ostreococcus exposed to light/dark cycles

**DOI:** 10.1186/1471-2164-11-192

**Published:** 2010-03-22

**Authors:** Annabelle Monnier, Silvia Liverani, Régis Bouvet, Béline Jesson, Jim Q Smith, Jean Mosser, Florence Corellou, François-Yves Bouget

**Affiliations:** 1 OUEST-genopole(R)transcriptome platform, IFR 140 GFAS, Faculté de Médecine, 2 avenue du Pr Léon Bernard, CS 34317 35043 Rennes Cedex, France; 2 Department of Statistics, University of Warwick, Coventry CV4 7AL, UK; 3 Medical Genomics Unit, Molecular Genetics and Biochemistry Department, Hospital Center, Rennes, France; 4 CNRS UMR 6061 "Génétique et Développement", Régulation transcriptionnelle et Oncogenèse, Université de Rennes 1, IFR140 GFAS, Faculté de médecine, 2 avenue du Pr Léon Bernard, CS 34317, 35043 Rennes Cedex, France; 5 UPMC University Paris 06, UMR7621 Laboratoire d'Océanographie Microbienne, Observatoire Océanologique, F-66651, Banyuls/mer, France; 6 CNRS, UMR7621 Laboratoire d'Océanographie Microbienne, Observatoire Océanologique, F-66651, Banyuls/mer, France

## Abstract

**Background:**

Picoeukaryotes represent an important, yet poorly characterized component of marine phytoplankton. The recent genome availability for two species of *Ostreococcus *and *Micromonas *has led to the emergence of picophytoplankton comparative genomics. Sequencing has revealed many unexpected features about genome structure and led to several hypotheses on *Ostreococcus *biology and physiology. Despite the accumulation of genomic data, little is known about gene expression in eukaryotic picophytoplankton.

**Results:**

We have conducted a genome-wide analysis of gene expression in *Ostreococcus tauri *cells exposed to light/dark cycles (L/D). A Bayesian Fourier Clustering method was implemented to cluster rhythmic genes according to their expression waveform. In a single L/D condition nearly all expressed genes displayed rhythmic patterns of expression. Clusters of genes were associated with the main biological processes such as transcription in the nucleus and the organelles, photosynthesis, DNA replication and mitosis.

**Conclusions:**

Light/Dark time-dependent transcription of the genes involved in the main steps leading to protein synthesis (transcription basic machinery, ribosome biogenesis, translation and aminoacid synthesis) was observed, to an unprecedented extent in eukaryotes, suggesting a major input of transcriptional regulations in *Ostreococcus*. We propose that the diurnal co-regulation of genes involved in photoprotection, defence against oxidative stress and DNA repair might be an efficient mechanism, which protects cells against photo-damage thereby, contributing to the ability of *O. tauri *to grow under a wide range of light intensities.

## Background

Photosynthesis by picophytoplankton (cyanobacteria and eukaryotic microalgae with a size < 2 μm) makes a significant contribution to global organic carbon production through carbon dioxide assimilation in the oceans. Eukaryotic picophytoplankton has a world- wide distribution and is an important contributor to biogeochemical cycles [1,2]. Of picoeukaryotes, three abundant ubiquitous genera from prasinophytes, *Ostreococcus*, *Bathycoccus *and *Micromonas *have been the most studied [3]. The first picoeukaryote to be identified was *Ostreococcus tauri*, a species initially identified as a major component of the picophytoplankton in the Thau lagoon [4]. *O. tauri *has been described as the smallest free-living eukaryote with the simplest ultrastructure that is one chloroplast, one mitochondrion, one Golgi body in addition to the nucleus.

In recent years, several genomes of *Ostreococcus *have been sequenced including *O. tauri *and *Ostreococcus lucimarinus *[5,6]. Two genomes of *Micromonas *became available recently [7] and a deep strain of *Ostreococcus *(RCC809) is now being sequenced. The analysis of *Ostreococcus *genomes has led to several hypothesis about physiological features, such as the occurrence of an atypical light harvesting complex and C4 photosynthesis [5,8]. An unusual high number of selenocysteine-containing proteins and a reduction of chromatin protein have been described in both strains of *Ostreococcus *but their significance is yet unknown [6]. Unusual features of *O. tauri *and *O. lucimarinus *genomes include high gene density, heterogeneous genome structure with two atypical chromosomes and high genome compaction. Analysis of gene content and evolution rates have suggested that lack of recombination and thus a lack of GC Biased Gene Conversion may be the origin of the lower GC content of the atypical chromosomes [9]. Phylogenetic footprints size distributions depend on gene orientation of neighboring genes and suggest a lower frequency of bidirectional regulatory elements in promoters in *Ostreococcus *as compared to budding yeast [10]. Clustering of genes involved in nitrogen assimilation on chromosome 10 suggest a possible link between gene localization on chromosomes and transcription. However all the above conclusions were inferred exclusively from *in silico *studies and the impact of genome structure on the transcription mechanisms remains to be addressed.

Little is known on the biology and physiology of eukaryotic picophytoplankton, which might explain their ecological success. In budding yeast, a genome-wide transcriptomic approach has revealed transcriptional networks associated with the temporal compartmentalization of cellular processes such as cell division during the metabolic cycle [11]. We have recently shown in *Ostreococcus*, that cell division is temporally regulated in cells exposed to diurnal cycle [12]. Most of the cell cycle regulators, including cyclin and cyclin-dependent kinase (CDK) family, were found to be transcriptionally regulated [12]. Therefore, to gain insight in the biology and physiology of *Ostreococcus *as well as the transcriptional basis of its genome structure, we chose to conduct a genome wide temporal analysis of gene expression in cells subjected to 12:12 day/night cycles. Under these conditions genes were found to be differentially expressed, most of them being regulated by the photoperiod. This regulation was observed for genes of both typical and atypical chromosomes. A Bayesian Fourier analysis revealed that more than 80% of differentially expressed genes had rhythmic patterns of expression. A detailed analysis of 2038 genes with strong diel rhythms of expression, yielded very well-defined time clusters with an abundance of genes involved in specific biological processes such as DNA replication, mitosis, translation, photosynthesis or lipid metabolism. Noteworthy, co-transcriptional regulations of genes involved in DNA repair and oxidative stress generated by light as well as photosynthesis and photoprotection were found. *Ostreococcus *contains less than 200 transcription factors [13], most of them being regulated by the photoperiod. This opens the way to a much fuller understanding of how coordinated transcriptional networks regulate biology and physiology in *O. tauri *and more generally in marine eukaryotic picophytoplankton.

## Results and Discussion

### Expressed genes show a differential expression over light/dark cycles

To identify genes with a diurnal rhythm, cells entrained under 12:12 light/dark (L/D) cycles were sampled every 3 hours for 24 hours with two overlapping time points at Time 9 (Light ON at Time 9; Light OFF at Time 21) in 3 independent experiments [12]. Under these medium light conditions (35 μmol quanta cm^-2 ^s^-1^), cell division is synchronized, occurring at the onset of night, and most of cell cycle genes are regulated by the diurnal cycle as checked by quantitative RT-PCR [12]. The expression of each time point was compared to a pool of all 3 kinetics 27 time points. Of the 8056 50 nt-long oligonucleotides probes, 7025 (80%) gave a median value of replicates 2.6 times above the background at least once (n = 27). The 981 probes exhibiting a signal below background may correspond to genes not expressed under our light/dark condition. For example, genes involved in sexual reproduction or in metabolic pathways active only under nutrient starvation are not expected to be expressed during exponential growth. It is also possible that some probes were designed against genes that were not correctly annotated in the automatic annotation of the genome or that for technical reasons some probes did not hybridize correctly to their targets.

**Figure 1:**
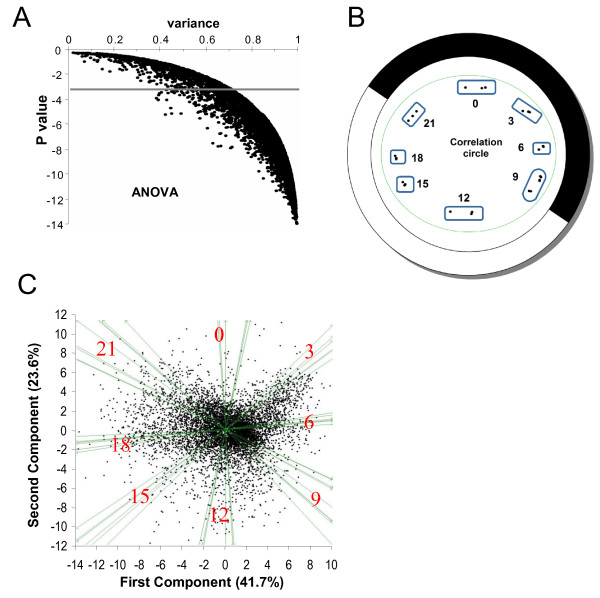
**Differential gene expression in *O. tauri *subjected to LD 12:12 cycles**. **(A) **ANOVA Analysis. 6822 probes detect genes with differential expression over 3 LD cycles with a Pvalue < 10^-3^. **(B) **Principal Component Analysis (PCA) of samples collected at different times of the LD cycle represented on a correlation circle. White and Dark represent light and dark period respectively (12:12; Light ON at Time 9; Light OFF at Time 21). Biological replicates are shown separately as dots. Note that the Time 9 overlapping time points are grouped. **(C) **Separation of genes by the first and second components of PCA shown on the x and y axis, which represent 41.7% and 23.6% respectively. The main axis partition morning/evening and night/day time points. At time 0, fewer genes are expressed.

A 3-factor ANOVA (probe, kinetics of expression and biological triplicate) identified 6822 probes corresponding to genes differentially expressed during the light/dark cycle with a Pvalue < 10^-3 ^(Fig 1A), among which 6610 probes gave a signal 2.6 fold over the background. Biological triplicates were highly reproducible as revealed by a principal component analysis (PCA) performed on the 27 time points and plotted on a correlation circle (Fig 1B). PCA on the individual 6822 gene probes confirmed that the differential gene expression over the time course (day/night, evening/morning) accounts most of the variability observed (Fig 1C). Fewer genes had their phase of expression 3 hours after dusk (Time 0) suggesting a gap in transcription at that time.

**Figure 2:**
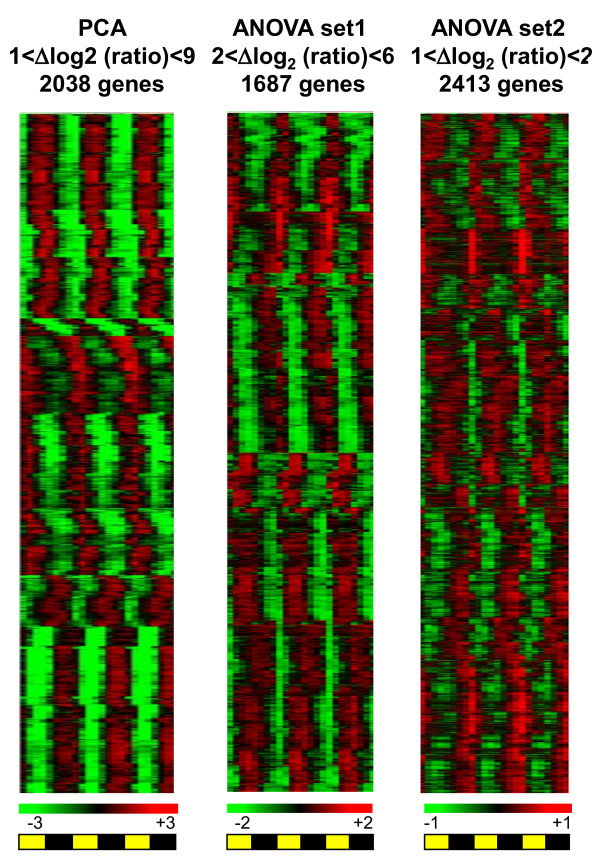
**Genome wide regulation of transcription by the photoperiod**. Hierarchical clustering of *Ostreococcus *genes according to their expression patterns. Because of the wide range amplitudes of amplitudes observed (1 <Δlog_2 _ratio <9) different sets of probes are represented: PCA set (2038 probes), ANOVA (non present in PCA) Set1 with 2 <lΔlog_2 _ratio <6 (1684 probes); ANOVA (non present in PCA) set 2 with 1 <lΔlog_2 _ratio <2 (2412 probes). Note that except for two, all genes with 6<Δlog_2 _ratio <9 are in the PCA set. High values of expression are represented in red, low values in green. The signals were normalized to the average level of the 3 L/D (12:12) cycles. Night periods are in black, light periods in yellow.

For subsequent analysis, we selected the 2038 gene probes with best dispersion after PCA as well as pools of genes selected according to their relative expression levels in the ANOVA set. Table 1 shows the number of gene probes, which exhibit diurnal changes according to their amplitude. Very high amplitudes in gene expression up to Δlog_2 _ratio = 9 were observed. Of 537 gene probes with Δlog_2 _ratio >5, 535 were selected after PCA and only 54 showed a two-to four fold induction (Table 1). In Figure 2, relative hierarchical gene expression is represented for three sets of genes, namely the PCA set (2038 probes), ANOVA set1 (2<Δlog_2 _ratio<6), ANOVA set2 (1<Δlog_2 _ratio<2). Waves of transcription were observed with phases corresponding to various times of the day over three LD. Globally all genes selected after ANOVA, had reproducible profiles of expression over three 12:12 LD cycles.

**Table 1:** Amplitude of cycling genes in 12:12 day/night cycles selected after ANOVA and Principal component analysis (PCA)

Δlog_2_(Ratio)	ANOVA set (6822 gene probes)	PCA set (2038 probes)
8-9	3	3
7-8	34	34
6-7	129	129
5-6	374	372
4-5	620	596
3-4	969	563
2-3	1544	287
1-2	2467	54
0-1	682	0

Amongst the TOP50 genes expressed with highest amplitude, three classes of genes were found (1) those involved in the cell division cycle, UV response and pigment biosynthesis, (2) those involved in metabolism including Krebs Cycle, (3) the last class contains and regulators of protein synthesis (Additional file 1). These genes encode mainly regulatory proteins whose expression is restricted to specific times of the day, such as CyclinB. In contrast, genes with the highest median expression values over the LD cycle are related to photosynthesis and ribosome structure (Additional file 2). These genes are well expressed housekeeping genes with low amplitude of expression or genes highly transcribed at specific times of the day such as the *ribulose 1,5 bisphosphate carboxylase *(*RubisCo*).

### Chromosomal patterns of transcription and genome structure

**Figure 3:**
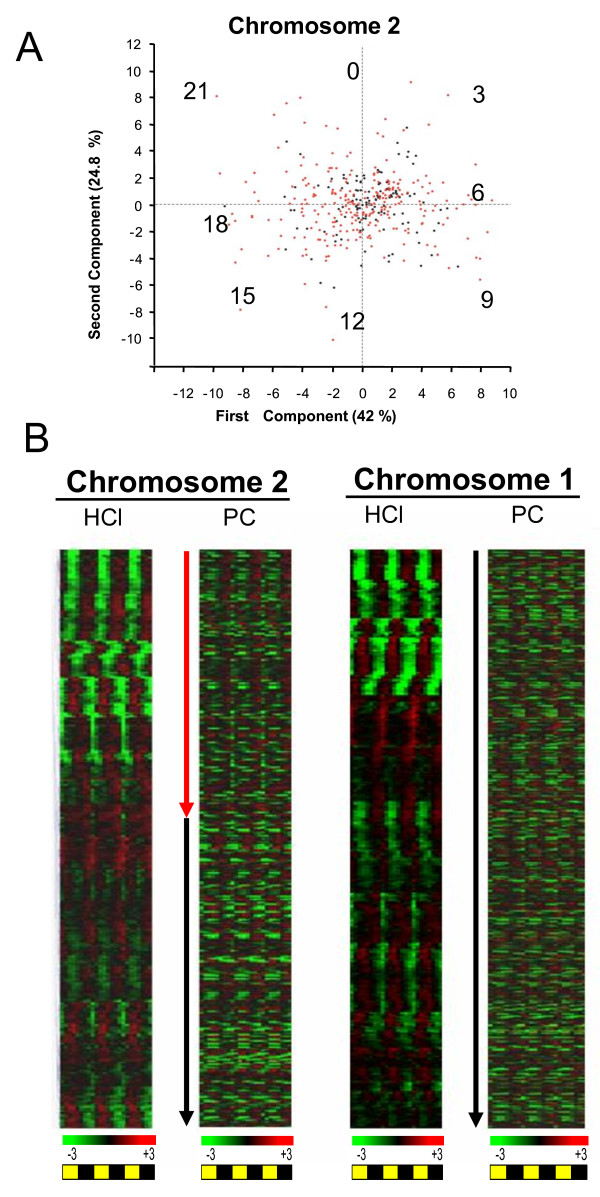
**Gene expression and genome heterogeneity**. **(A) **Principal Component Analysis on genes located on atypical chromosome 2. Atypical genes from the first half of the chromosome are plotted in red, typical genes from the second part are plotted in black. The first and second components shown on the x and y axis, represent 42% and 24.8% of total variation respectively, very similar to the other chromosomes. **(B) **Hierarchical clustering (HCl) of gene expression on atypical Chr2 compared to Chr1. Expression of genes is also represented according to the physical localisation along chromosomes (PC). Top correspond to 0 position of the Chromosome.

Both *Ostreococcus *genomes present unique features such as gene compaction with intergenic regions below 200 bp in size and several examples of gene fusions. Genome heterogeneity was reported in both *O. tauri *and *O.lucimarinus *genomes. In *O. tauri*, chromosome2 (Chr2) and chromosome19 (Chr19) have a significantly biased G+C content, unusual introns and contain most of the transposons-like elements. Furthermore these chromosomes in both species show lower levels of synteny and different gene densities compared to the other chromosomes [6]. Because of their structure, genes on atypical chromosomes are good candidates for recent horizontal gene transfer from bacteria into *Ostreococcus *[6] In a such a scenario, genes located on chromosome regions of bacterial origin may have kept signatures of prokaryote transcription, which is organized in operons. We focussed on the transcription patterns of genes located on atypical chromosomes (Figs 3A and 3B). Day/night oscillations of transcript levels were observed for genes of typical chromosomes such as Chr1) and atypical chromosomes such as Chr2 as shown in Hierarchical clustering (HCl) (Fig 3B). PCA on Chr2 yielded a similar day/night partitioning as for the whole genome (Fig 3A) and did not reveal heterogeneity in gene expression between the genes from the two halves of Chr2. Overall, clustering of transcription patterns based on chromosome localisation (PC) did not reveal major differences between the transcription patterns of genes localized on atypical chromosomes and other chromosomes (Fig 3B). Therefore, if genes belonging to atypical chromosomes are of prokaryote origin, they must have fully integrated eukaryotic mechanisms of transcription, so that they are transcribed as autonomous units. Operons have no been identified in *Ostreococcus*, however genes involved in nitrate, ammonium and urea clustered in a chromosome region of 19 kpb [5]. Patterns of transcriptions of this chromosome area covered different phases of the day, indicating that these genes are not transcriptionally coregulated. Together our data did not reveal any obvious link between gene localization on chromosomes and the photoperiod-regulated transcription in *O. tauri*, suggesting that both typical and atypical chromosomes have canonical eukaryotic mechanisms of transcription.

The genes located on the lower G+C content regions of Chr2 and Chr19 have been shown to evolve significantly faster than the other genes[9]. This may be the consequence of lack of recombination or increased mutation rate on these chromosomes. Transcription has been shown to be mutagenic in some species. Genes located on atypical chromosomes with low GC contents, did not exhibit unusual mechanisms of transcription, suggesting that transcriptional induced mutation bias is unlikely to be the origin of the lower GC content of these chromosomes.

In summary, despite the compactness and heterogeneity of its genome, *O. tauri *does not appear to exhibit unusual mechanisms of transcription related to its genome structure.

### Bayesian Fourier analysis reveals rhythmic patterns of expression associated with specific biological processes

A Bayesian clustering method based on Fourier coefficients allowed us to discriminate putative regulatory genes [14]. This method is well-understood, rapid, and flexible [15]. The Fourier coefficients capture the rhythmic properties of interest to us by measuring the contribution of sine and cosine waves with differing periods to the rhythmic patterns in the data. We used an agglomerative hierarchical algorithm for clustering of gene expression patterns, based on the Fourier coefficients (see Materials and methods). This method discriminates among rhythmic patterns based on the amplitude and waveform, in addition to the phase. Photoperiod-regulated expression profiles were identified by the dominant contribution of the sine and cosine waves with a 24-h period. For computational reasons, only the first, third, sixth and ninth harmonics, along with the constant term, were included, yielding 9 parameters. Moreover, a direct BFC analysis could not be performed on all 6822 differentially expressed genes due to memory space limitation. Therefore, three pools of randomly selected genes were generated for BFC analysis and analyzed separately.

BFC discriminates patterns based on waveform, phase and amplitude. In our analysis, the third harmonic ratio (THR) was chosen to assess rhythmicity (see Materials and Methods). Clusters were scored as rhythmic for THRs value above 0.4 [14]. We identified out of 489 clusters, 433 with a THR above 0.4 corresponding to 5977 probes (86% of the probes). We therefore concluded that genes identified as differentially expressed correspond to rhythmically expressed genes. Genome wide regulation of gene expression by the photoperiod has been described in cyanobacteria [16]. In the unicellular eukaryotic green alga *Chlamydomonas*, only 2.6% of the genes were shown to be under circadian control [17]. Several studies in diatoms have reported global transcriptome changes in response to iron or silicon starvation [18,19], however our study is the first example of a global regulation of transcription by the photoperiod in eukaryotic phytoplankton. Such a global rhythmicity of transcription resembles the waves of transcription observed during the metabolic cycle of budding yeast [11]. In the plant *Arabidopsis *more than 30% of the transcripts were shown to be regulated by the photoperiod [20] and enhancer trap suggests that 36% of the genes are under circadian control [21]. A recent study has revealed that 89% of *Arabidopsis *genes cycle in at least one condition of LD, circadian or thermocycles [22]. In our single LD 12:12 condition, expressed genes in *O. tauri *display rhythmic expression patterns over the time, consistent with a global regulation of transcription under light/dark cycles.

**Figure 4:**
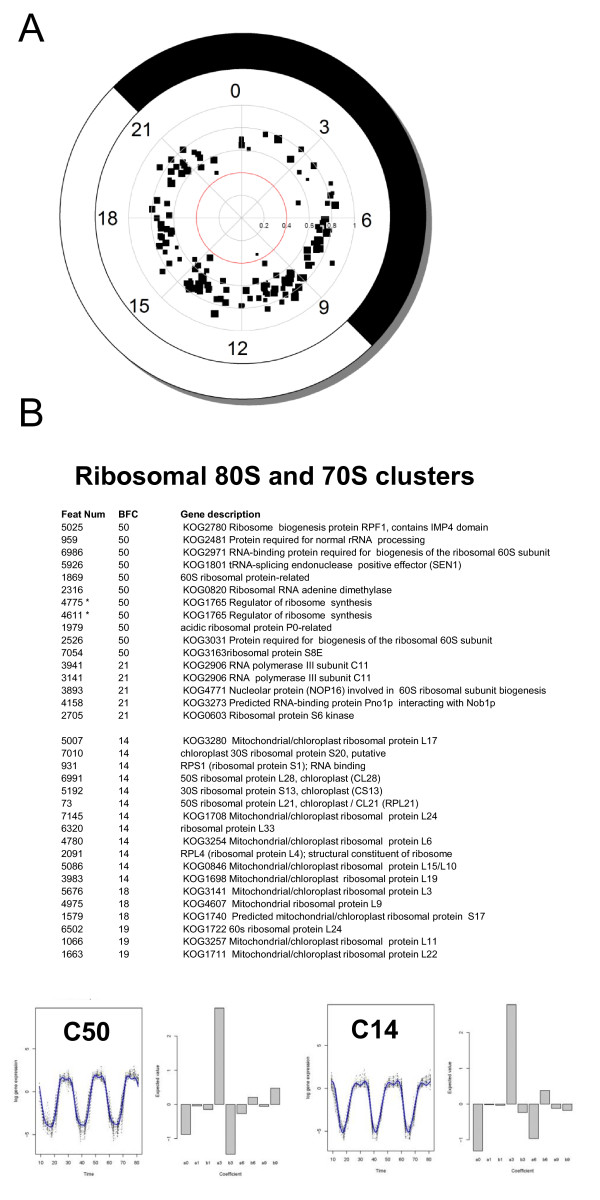
**Bayesian analysis reveals group of coregulated genes associated with specific biological processes under LD cycles**. **(A) **BFC clusters from 2038 gene probes selected after PCA. In the polar plot each squared dot represents a cluster, with the size of the dot proportional to the size of the cluster. The clusters are positioned around the clock depending on when they peak during the day (Light and dark period are represented in white and black respectively). The distance from the centre is greatest when the value of the THR - measuring the diurnal component of the expression - is greatest. **(B) **Examples of 70S ribosome and 80S ribosome gene clusters. Feature Number (Feat Num), BFC cluster number (BFC). Stars indicate two probes corresponding to two different feature numbers associated to the same gene in the final annotation. The graphical output consists of a plot, on the right hand side, containing the profiles of the genes that belong to that cluster, together with the estimated posterior mean for that cluster, and a bar plot, on the right hand side, showing the expected posterior values β. Note that cluster 50 and 21 as well as clusters 14, 18 and 19 have nearly identical profiles and for convenience only one cluster profile (e.g. C50) is shown.

A first analysis of BFC clusters did not allow the identification of clusters associated with specific biological processes. We therefore decided to focus on the genes with robust rhythms of expression selected after PCA. A large number of clusters (138) was generated and the size of each cluster was relatively small (2 to 50 probes). Only one cluster containing 2 probes had a THR value under 0.4 and 1893 probes belong to clusters with THR values above 0.6, confirming that the selected genes had robust rhythms of expression. Consistently with the results of the PCA (Fig 4A), fewer genes fell in BFC clusters around time 0 confirming a gap in transcription at this time of the day. Because the size of the clusters was small, each cluster was examined individually. For this analysis we used the annotation of *Ostreococcus *genome primarily based on KOGG classes together with an annotation based on *Arabidopsis *non redundant database (see Materials and methods). BFC clustering revealed biological processes associated with specific clusters. Transcriptional coregulation of genes encoding mitochondrial/plastidial ribosomal protein is one the most striking example of a transcriptional network regulated by the photoperiod (Fig 4B). For example, cluster 14 contains 26 probes of which eleven encode 70S plastid/mitochondria ribosomal protein. Cluster 18 (9 probes), which has an almost identical profile as cluster 14 has 3 plastid/mitochondria proteins and a chloroplast related IF2 translation initiation factor. Several genes involved in 80S ribosome biogenesis including RNA polymerase III, were overrepresented in Clusters 50 and 21 (Fig 4B).

**Figure 5:**
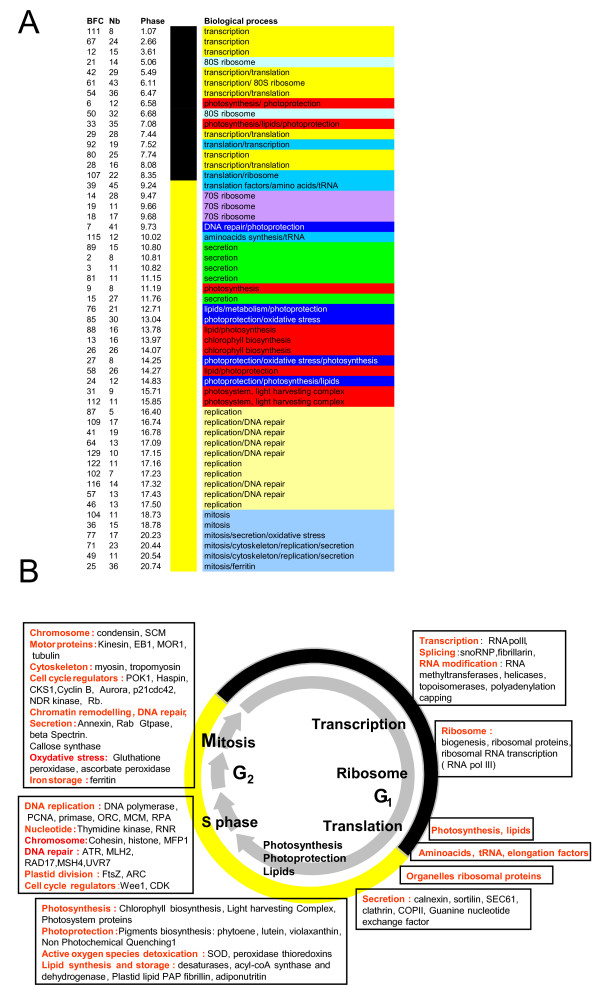
**Sequential expression of genes associated with specific biological processes under LD cycles**. **(A) **Main clusters associated with biological processes, plotted according to their phase of expression. BFC cluster (BFC), number of gene probes in the cluster (Nb) and Phase of expression (Phase). Biological processes are represented by different colours. Black and yellow vertical bands represent night and light periods respectively. **(B) **Overview of the transcriptional regulations of the main biological processes during a light/dark cycle. During the night actors of transcription, translation and protein synthesis are sequentially transcribed. During the light period, genes of Photosynthesis and lipid metabolism are transcriptionally coregulated. DNA repair and photoprotection genes are found in midday clusters. At the end of the day, specific transcriptional networks are associated with DNA replication and mitosis.

Classification of gene clusters according to their phase of expression (Fig 5A) revealed that transcripts involved in specific biological processes, were associated with certain time intervals during the day. For example, most genes implicated in protein synthesis had their phase of expression around dawn while replication genes peaked 3 hours before night and mitosis genes at dusk (Fig 5A). To our knowledge, such a coregulation of biological processes has never been observed to this extent in eukaryotic cells exposed to 24 h light/dark cycle. This gave us a unique opportunity to obtain insight into the biology and physiology of *O. tauri *based on transcription profiling of the genome expression under light/dark conditions where most genes are expressed.

### The sequential transcription of genes involved in RNA synthesis/processing and ribosome biogenesis during the night, translation and amino-acid synthesis at dawn

Midnight clusters such as 111, 67 and 12 contain predominantly genes involved in basic transcription machinery, however the main transcription clusters peaked in late night between time 5 and 8 (Figs 5A and 5B). They include RNA polymerase II, splicing (U4/U6 small ribonucleoprotein Prp4, splicing factor PRP31), mRNA cleavage and polyadenylation factor, polyA binding protein, nuclear cap binding proteins as well as RNA helicases, RNA methyltransferases and DNA topoisomerases (Additional file 3). Most transcription clusters had similar profiles, although some small differences were observed such as between clusters 111/67, 61/54 and 29/42. The genes encoding proteins involved in 60S cytoplasmic ribosome biogenesis and ribosomal RNA (RNA pol III) peaked 3 hours before light ON in two clusters with nearly identical shapes (Clusters50/21), suggesting that ribosomal proteins and ribosomal RNA are transcriptionally coregulated (Fig 5A, B, see also Fig 4A). This contrast with the large clusters of genes involved in the biogenesis of 70S ribosome, which are expressed 3 hours after dawn, and suggests that the translational processes might be temporally uncoupled in the cytoplasm and the organelles, the second occurring during the light period. Interestingly, 18 plastidial ribosomal genes were reported to be under circadian control with a similar morning phase in *Chlamydomonas *[17].

Many genes involved in the regulation of translation, tRNA and aminoacid synthesis had a phase of expression at dawn (Fig 5; Additional file 4). Six regulators of translation, including Translation initiation eIF-4G, eIF-3, eIF-3f, the translational repressor Pumilio/PUF3 and related RNA-binding protein and translation ribosome release factor APG3 as well as tRNAs were mainly found in clusters 39/10 7 and 92, suggesting further a possible regulation of protein synthesis at dawn.

It is tempting to speculate that the sequential expression of transcription and translation basic machinery, after a gap in global transcription early in the night, may anticipate dawn to ensure a tight regulation of protein synthesis from that time.

### DNA replication and DNA repair genes are coregulated

In *Ostreococcus*, cell division is synchronized by the photoperiod and several S phase genes were shown to be express at the end of the light period [12]. Ten clusters with phases between time 16.5 and 17.5 were enriched in genes related to DNA replication (Fig 5; Additional file 5). The Proliferating Cell Nuclear Antigen (PCNA), a well known S phase marker, was found in these clusters. Within these 50 S-phase genes, a majority (33) was related to DNA replication, including sister chromatin cohesion proteins, MCM and Origin Recognition complex (ORC), DNA polymerase and Ribonuclease H1. DNA replication clusters contained also thymidine kinase and ribonucleotide reductase (RNR), which are involved in nucleotide synthesis. Interestingly genes involved in DNA mismatch repair from MLH2/PMS1/Pms2 family, ATR/Tel1 kinase involved in DNA damage signalling or RAD17 had similar transcript profiles as DNA replication genes. Like many phytoplanktonic species *Ostreococcus *cells are exposed to DNA damage due to UV exposure. In our experiment cells were grown at relatively low light intensity (35 μmol quanta cm^-2 ^s^-1^) lacking UV. It is therefore likely that the transcription of DNA repair genes is directly regulated by the photoperiod and/or by the circadian clock rather than by light intensity. Such a mechanism would be efficient for anticipating photo-damage and repairing DNA upon UV exposure during the day.

Several genes involved in chloroplast division such as *FtsZ *and *ARC5 *were also detected in S phase clusters. Such a regulation might ensure the coordination of nuclear and chloroplast division, which takes place during nuclear DNA replication.

### Oxidative stress defence and photoprotection genes

We were unable to identify any conventional catalase in the genome of *O. tauri *suggesting that *Ostreococcus *uses an alternative mechanism to detoxify reactive oxygen species. Several genes involved in oxidative stress defence (thioredoxins) and against damaging light environments (Non Photochemical quenching 1) were found in cluster 85, 3 hours after dawn (Fig 5; Additional file 6). Likewise the transcriptional coregulation of UVR3-6-4 photolyase involved in DNA repair upon UV exposure and the copper/zinc superoxide dismutase known to detoxify reactive oxygen species (cluster 27) might be involved in protection against photo-damage.

Carotenoids pigments including violaxanthin, zeaxanthin and lutein, which protect cells from photo-oxidative damage, have been described in *O. tauri *[23]. Interestingly, phytoene desaturase, a precursor enzyme in carotenoid biosynthesis upstream of violaxanthin and zeaxanthin synthesis and zeaxanthin epoxidase, violaxanthin-de-epoxidase and Cytochrome P450 reductase is in cluster7, which also contains enzymes involved in DNA repair such as formamidopyrimidine-DNA glycosylase (Additional file 6). Our light intensity condition is more than 10 times lower than intensities *O. tauri *can survive [23]. Therefore, it is likely that genes involved in photoprotection and defence against oxidative stress generated by light are directly regulated by the photoperiod or by the circadian clock rather by light intensity. This regulation would allow *O. tauri *to anticipate predictable daily high light intensities encountered in the environment and account for its capacity to grow under relatively high light intensities [24].

### Coregulation of genes involved in mitosis

Mitosis is atypical in *O. tauri *since the nuclear envelope does not break down, no chromosomes have been observed and at the most two microtubules were seen using electron cryotomography [25]. The cell division cycle *per se (*SG2 M) is short in *O. tauri *and lasts less than 3 hours leading to a partial overlap of cell cycle phases in cell populations synchronized by light/dark cycles [26]. However at time 12 most of the cell undergo cytokinesis as cell number is increasing and the proportion of G2 M cells is decreasing compared to G1 cells. Consistently, several clusters (36, 62, 71 and 77) peaking at time 12 contained mitotic genes such as chromosome condensation complex condensins and Structural Maintenance of Chromosome2 (SMC2), a gene required for chromosome segregation (Fig 5; Additional file 7). Tubulin, including gamma-tubulin, and microtubule associated motor proteins such as kinesins were expressed at the time of mitosis, even though a mitotic spindle has never been observed in *Ostreococcus*. Well known regulatory proteins of mitosis such as B-type cyclin, CDK subunit1, the Haspin mitotic histone kinase required for metaphase chromosome alignment or the mitotic checkpoint kinase Bub1 and Aurora kinase were also identified in these clusters.

Again, two genes encoding peroxide detoxifying enzymes ascorbate and gluthatione peroxidases were transcribed together with mitotic genes suggesting that they might be important to protect DNA against oxidative damage at the time of division.

Surprisingly ferritin was maximally expressed at the time of mitosis. In the oceans iron is often a limiting factor for phytoplankton growth and ferritin was shown to confer an ecological advantage to pennate diatoms [27]. In *Ostreococcus *ferritin might be important for iron storage at the end of the day since iron is found mainly associated to the photosynthetic apparatus in the chloroplast during the day.

The presence of several genes involved in Golgi-derived secretion such annexin, spectrin and callose synthase in mitotic clusters suggests that they might be related to the massive secretion of Golgi-derived material observed at the time of cell division. The nature of secreted molecules at the time of division is unknown but the secretion of polysaccharides such as callose might be linked to the absence of cell wall in *Ostreococcus*. Several clusters enriched in secretion genes such as guanine nucleotide exchange factor, vesicle coat complex COPII or protein transport SEC61 had an earlier phase around time 3 (Fig 5). Whether their expression is related to cell cycle progression remains to be determined.

### Coregulation of photosynthesis and lipid metabolism genes

Aside two nearly identical clusters (C33/C6) with a late night phase of expression at 7 hours after light ON, other photosynthesis clusters had a mid-day phase of expression (Figure 5; Additional file 8; Additional file 9). C33/C6 late night clusters contained genes involved in chloroplast biogenesis (GcpE chloroplast biogenesis4; chloroplast Biogenesis 6), photosystem I assembly protein Ycf4 and precursors of chlorophyll and carotenoid biosynthesis such as geranylgeranyl reductase (Additional file 8). Interestingly, these clusters were also enriched in genes related to lipid biosynthesis and storage such as phosphatidylinositol transfer protein, delta 6-fatty acid desaturase/delta-8 sphingolipid desaturase, delta12-fatty acid dehydrogenase desaturase, Fatty acid biosynthesis I, oxysterol binding protein or patatin. Genes belonging to Calvin cycle (phosphoribulokinase), glycolysis (fructose-bisphosphate aldolase, triose phosphate isomerase) and pentose phosphate pathway (Ribose-phosphate pyrophosphokinase) were also identified in these clusters. These genes may be mainly under circadian rather than direct light control since their expression anticipates dawn. Such a mechanism might be used to optimize light assimilation from dawn. The soluble starch synthase III (SSIII), a key enzyme involved in the synthesis of the long glucan fraction, is required for circadian rhythm of starch content, which peaks in the middle of the night phase in *Chlamydomonas *[28]. The photoperiod/circadian regulation of starch content is currently unknown in *Ostreococcus*. Based on the phase of *SSIII *transcript(cluster33), it would not be surprising to find a circadian regulation of starch content in *Ostreoccocus *like in *Chlamydomonas*.

On the other hand many genes involved in chlorophyll biosynthesis and light harvesting complex genes were found in separate well defined clusters with an afternoon phase at Time 14 (Additional file 9) suggesting that they are controlled by distinct transcriptional networks.

### Transcriptional regulation of transcription factors

A vast majority of *Ostreococcus *genes appear to be regulated by the photoperiod. Only 183 transcription factors were identified in *O. tauri*, amongst which 170 were expressed. Like other genes, they had reproducible rhythmic patterns of expression, with all phases of the day being represented (Additional file 10). Transcription factors such as MAD-Box being present as a single member were expressed only at certain times of the day. Assuming that transcript levels reflect the level of protein, this would suggest that either their activity is restricted to specific times of the day or that their protein are present at steady state levels, which are not correlated to the level of transcripts. In case of multigenic families, such as HMG all phases of expression were observed. Interestingly, 5 out of 7 CCAAT-HAP3 and CCAT-HAP5 had very similar pattern of expression, peaking at the end of the day, suggesting that CCAT-dependent transcription may be more active at this time of the day. Comparisons of transcription patterns of genes containing CCATT boxes in their promoter may help to address this question.

## Conclusions

Most of expressed genes of *O. tauri *appear to be transcriptionally regulated under light/dark cycles and display robust rhythms of expression. In addition, high resolution Bayesian Fourier Clustering analysis revealed the occurrence of transcriptional networks associated with specific biological processes such as transcription, translation, photosynthesis and cell division. This should allow the identification of new genes involved in specific processes or interconnected transcriptional networks. For example the coregulation of genes involved in DNA replication, DNA repair and photoprotection may account for the ability of *O. tauri *to grow under a wide range of medium to high light intensities. Together the limited set of transcription factors, the small size of intergenic regions and the availability of sequences of several *Ostreococcus *ecotypes, should make possible to identify response elements in promoters of coregulated genes. Genetic transformation was recently developed and used to characterize a conserved circadian Evening Element in the promoter of the *Ostreococcus Time of CAB Expression-1 *clock gene [29]. Future transcriptomic studies coupled to phylogenetic footprint and functional analysis should give insight into the transcriptional networks involved not only in diurnal regulation of gene expression but also in response to specific stresses of the marine environment such as phosphate, nitrogen or iron limitation, UV stress or in response to viral infection.

## Methods

### Slides construction and hybridization

Genome-wide based *Ostreococcus *slides (24 K) were manufactured in the Genopole Ouest Transcriptome Platform (Rennes, France). Gene-specific 50-mer oligonucleotides (8,056) were designed and synthesized by Eurogentec on the basis of January 2006 annotation. In the final annotation of the genome (June 2006, http://bioinformatics.psb.ugent.be/blast/public/?project=ostreococcus), 6369 genes were represented by at least one probe (5435 by a single probe, 791 by two probes, 116 by 3 probes and 27 by more than 4 probes) but 565 oligonucleotides did not match the genome anymore in BlastN. However 372 out these 565 probes gave a good and reproducible hybridization signal, were selected after ANOVA as differentially expressed genes (see below). Therefore each probe was attributed a feature number with corresponding numbers in the two annotations. Cell culture conditions, RNA extraction, labelling, hybridization and raw analysis have been previously described [12].

### Microarray data analysis

Normalization was performed using the print-tip loess method and scaled with the Gquantile method [30,31]. Time courses of gene expression were performed in triplicate, over 27 h, at 3 h intervals (nine time points per time course). Fifteen probes, where on more than 70% time points no data are available, were removed from the analysis. We first verified hybridization robustness by performing a hierarchical clustering on the 8041 selected probes using TiGRMeV4.0 suite [32]. Technical triplicates were clustered. Therefore, for further analysis, we chose to work on the median value of each technical triplicate.

Analysis of Variance (ANOVA) and Principal Component Analysis (PCA) were performed using the GeneANOVA software [33] and the limma (R package) from bioconductor [34]. 6822 genes differentially expressed with a P value < 10-3 were selected using a 3 factors (genes, biological kinetics and biological replicates) ANOVA. Correlations were found between gene expression and time points with PCA and we retained 2038 genes with best dispersion corresponding to maximized variance. Twelve gene expression clusters were highlighted with SOM 2D (Self organizing Map) provided in TiGRMeV4.0 suite (Current metric = pearson correlation) and analysed using FATIGO based on *Arabidopsis *functional annotation. Qualitative information was obtained about biological processes associated with specific times of the day. However, only a small number of homologues of *Arabidopsis *annotated genes were found. For this reason, these clusters were not further analyzed.

Bayesian Fourier Clustering (BFC) was used to cluster time series according to their expression profiles using the framework of a standard linear model [14]. Curves were clustered together by BFC if they appeared to have been drawn from a joint distribution with parameters β and σ^2^, where Y = Bβ + ε and Y represents the logarithm of the expression levels. ε is a noise term, which is normally distributed with mean zero and variance σ^2^. Thus the skewed time course of expressions of genes in each cluster is characterized by a different vector of Fourier coefficients β and associated variance σ^2^. This technique is therefore a powerful way of uncovering a wide variety of shapes and respects the time ordering of expressions. This method was exceptionally fast because of the choice of distributions on the parameters [35], the settings of the hyperparameters [15] and the hierarchical search among partition spaces. Each gene expression profile was initially assigned to an individual cluster. Then the two clusters most similar in covariance structure were merged to produce a new set of clusters. The process on the current set of clusters was repeated until all profiles lie in a single cluster. At each merger, the clustering was scored; the highest score was obtained for a partition of the 2038 gene probes into 138 clusters. Our flexible C++ code enabled us to apply the above model to the *O. tauri *data without the multistage clustering, which can cause the undesired loss of genes [14]. The design matrix B was customised and chosen to contain Fourier basis functions to help in the identification of rhythmic genes. The vector β holds the Fourier coefficients for the average profile of each cluster. For computational reasons and given the nature of the data, only the first, third, sixth and ninth harmonics, along with the constant term, were included. These values produced the average profile seen as the blue line in Figure 4(B) and in the Supplementary Figures. Clusters were identified by dominance of the harmonic associated with a high 24-hr diurnal component relative to other harmonics. Adapting a statistic used in Edwards *et al*. (2006) we measured the strength of the diurnal variation of the 3-day experiment by the third harmonic ratio (THR): THR =  where *a*_i_is the coefficient of the *i*-th cosine term, *b*_i _the coefficient of the *i*-th sine term and *i *indexes the particular harmonic.

Genes in the final KOG based genome annotation (June 2006) corresponding to *O. tauri *probe sequences were blasted (BLASTX) on *Arabidopsis *non redundant database to identify *Arabidopsis *closest orthologue. Identification of biological processes associated with BFC clusters was done on the basis of both annotations.

The complete dataset has been submitted to the Gene Expression Omnibus (GEO) public database at NCBI under the accession number: GSE16422. (Processed data: http://www.ncbi.nlm.nih.gov/geo/query/acc.cgi?token=jbmhpwkyccmuwvu&acc=GSE16422

## Authors' contributions

AM analyzed microarray data, performed statistical analysis using ANOVA and PCA and she was also involved in writing of the manuscript and submitting data to GEO. SL implemented BFC analysis for the *Ostreococcus *dataset, designed BFC clustering and was involved in writing. RL participated to microarray and statistical analysis. BJ improved the annotation of *Ostreococcus *genes, based on existing annotations and databases search. JS participated to BFC analysis. FC was involved in manual analysis of BFC clusters. FYB coordinated the work and was more specifically involved the manual analysis of BFC clusters and writing of the manuscript. All authors read and approved the final manuscript.

## Supplementary Material

Additional file 1 **List of TOP50 genes with highest amplitude**. TOP50 genes ranked according to their amplitude as log_2_(ratio). Click here for file

Additional file 2 **List of Top 50 genes with highest median expression**. TOP50 genes ranked according to their median hybridization signal (absolute value). Click here for file

Additional file 3 **Coregulation of genes involved in basic transcription machinery during the night**. BFC clusters from 2038 gene probes selected after PCA. Each colour corresponds to a biological process. Feature Number (Feat Num), BFC cluster number (BFC). Stars indicate two probes corresponding to two Feature Numbers associated to a single gene in the final annotation. Bottom right: The main BFC profiles and coefficients are shown. Note that clusters 61, 54, 111 and 67, clusters 29 and 92 as well as clusters 80 and 28 have almost identical profiles and for convenience only one profile (e.g. 61) is shown. Click here for file

Additional file 4 **Clusters of genes involved in protein synthesis including translation regulators, tRNA and amino acid biosynthesis around dawn**. BFC clusters from 2038 gene probes selected after PCA. Each colour corresponds to a biological process. Feature Number (Feat Num), BFC cluster number (BFC). Right: The main BFC profiles are shown. Note that clusters 39 and 107 have nearly identical profiles. Click here for file

Additional file 5 **Coregulation of DNA replication and DNA repair genes at the end of the light period**. S Phase BFC Clusters from 2038 gene probes selected after PCA. Each colour corresponds to a biological process. Feature Number (Feat Num), BFC cluster number (BFC). Right: The main BFC profiles and coefficients are shown. Note that clusters 41, 64 and 87 as well as clusters 57 and 116, clusters 46, 122 and 129 have nearly identical profiles. Click here for file

Additional file 6 **Cluster of genes involved in photoprotection, defence against oxidative stress and DNA repair around midday**. BFC clusters from 2038 gene probes selected after PCA. Each colour corresponds to a biological process. Feature Number (Feat Num), BFC cluster number (BFC). Right: The main BFC profiles and coefficients are shown. Click here for file

Additional file 7 **Coregulation of genes involved in mitosis at dusk**. Mitotic BFC clusters from 2038 gene probes selected after PCA. Each colour corresponds to a biological process. Feature Number (Feat Num), BFC cluster number (BFC). Right: The main BFC profiles and coefficients are shown. Note that clusters 71 and 49 as well as clusters 104 and 36 have nearly identical profiles. Click here for file

Additional file 8 **Late night clusters of genes involved in chloroplast biogenesis, pigment biosynthesis, lipid biosynthesis and metabolism**. BFC clusters from 2038 gene probes selected after PCA. Each colour corresponds to a biological process. Feature Number (Feat Num), BFC cluster number (BFC). Right: The main BFC profiles and coefficients are shown. Note that clusters 33 and 6 have nearly identical profiles. Click here for file

Additional file 9 **Afternoon genes involved in Chlorophyll and Photosystem proteins biosynthesis**. BFC clusters from 2038 gene probes selected after PCA. Each colour corresponds to a biological process. Feature Number (Feat Num), BFC cluster number (BFC). Stars indicate two probes corresponding to two Feature Numbers associated to a single gene in the final annotation. Right: The main BFC profiles and coefficients are shown. Click here for file

Additional file 10 **Transcriptional regulations of transcription factors**. (A) Hierarchical clustering of 170 expressed transcription factors. (B) Transcription patterns of expression of CCAAT HAP3/HAP5 transcription factors. Click here for file
