# Supplementary material for: Orchestrated transcription of biological processes in the marine picoeukaryote Ostreococcus exposed to light/dark cycles
Source: BMC Genomics. 2010 Mar 22;11:192. doi: 10.1186/1471-2164-11-192 (PMC2850359; doi:10.1186/1471-2164-11-192)
Supplement: Additional file 1 — List of TOP50 genes with highest amplitude. TOP50 genes ranked according to their amplitude as log2(ratio). [file 1471-2164-11-192-S1.PDF]

Additional data file 1: TOP50 genes with highest amplitude

Feat Num	Gene description	Chr
4751	KOG0266 WD40 repeat-containing protein	15
3798	KOG0385 Chromatin remodeling complex WSTF-ISWI, small subunit	9
6245	KOG0471 Alpha-amylase	16
8051	KOG0968 DNA polymerase zeta, catalytic subunit	8
7189	KOG2957 Vacuolar H+ATPase V0 sector, subunit d	8
7054	ribosomal protein S8e family protein	17
6412	KOG0996 Structural maintenance of chromosome protein 4	6
2316	dimethyladenosine transferase, putative	8
1710	PGM (PHOSPHOGLUCOMUTASE)	15
3897	UVR3 (UV REPAIR DEFECTIVE 4)	5
7883	KOG0501 K+channel KCNQ	9
4921	KOG1496 Malate dehydrogenase	6
1339	KOG2708 Predicted metalloprotease with chaperone activity	5
6901	regulator of chromosome condensation (RCC1)/ UVB-resistance protein	5
7926	KOG2019 Metalloendoprotease HMP1 (insulinase superfamily)	6
305	KOG2807 RNA polymerase II transcription initiation	6
1047	KOG1404 Alanine-glyoxylate aminotransferase AGT2	20
4611	KOG1765 Regulator of ribosome synthesis	15
7194	KOG3111 D-ribulose-5-phosphate 3-epimerase	6
3886	KOG2335 tRNA-dihydrouridine synthase	8
7391	ribosomal protein L29 family protein	6
7860	KOG0800 FOG: Predicted E3 ubiquitin ligase	10
4749	KOG1441 Glucose-6-phosphate/phosphate antiporter	15
7597	KOG2323 Pyruvate kinase	16
5197	flavin reductase-related	4
5030	FFC (FIFTY-FOUR CHLOROPLAST HOMOLOGUE)	1
25	GUN4 (Genomes uncoupled 4)	17
4186	MATE efflux family protein	4
4886	lipase class 3 family protein	6
5756	KOG1248 Uncharacterized conserved protein	15
6434	KOG2974 Uncharacterized conserved protein	18
3200	KOG1256 Long-chain acyl-CoA synthetases (AMP-forming)	16
267	KOG4153 Fructose 1,6-bisphosphate aldolase	10
6260	KOG1601 GATA-4/5/6 transcription factors	13
1865	CHLD/PDE166 (PIGMENT DEFECTIVE 166)	5
6336	KOG0054 Multidrug resistance-associated protein	3
2769	KOG1282 Serine carboxypeptidases (lysosomal cathepsin A)	10
5347	KOG4747 Two-component phosphorelay	8
6458	EMB2726 (EMBRYO DEFECTIVE 2726); translation elongation factor	4
3537	DIT1 (DICARBOXYLATE TRANSPORTER 1); oxoglutarate:malate antiporter	4
1727	KOG0358 Chaperonin complex component, TCP-1 delta subunit (CCT4)	12
2915	DIT2.2 (DICARBOXYLATE TRANSPORTER 2.2)	4
3775	PDS3 (PHYTOENE DESATURASE)	10
1967	KOG0653 Cyclin B and related kinase-activating proteins	1
5025	KOG2780 Ribosome biogenesis protein RPF1	1
5055	KOG1112 Ribonucleotide reductase, alpha subunit	1
6370	phosphoglycolate phosphatase, putative	8
931	RPS1 (ribosomal protein S1); RNA binding	2
1979	acidic ribosomal protein P0-related	17

Cell division, protein synthesis , metabolism, photoprotection.
Chr (chromosome number), Feat Num (Feature Number)
