# Supplementary material for: Orchestrated transcription of biological processes in the marine picoeukaryote Ostreococcus exposed to light/dark cycles
Source: BMC Genomics. 2010 Mar 22;11:192. doi: 10.1186/1471-2164-11-192 (PMC2850359; doi:10.1186/1471-2164-11-192)
Supplement: Additional file 2 — List of Top 50 genes with highest median expression. TOP50 genes ranked according to their median hybridization signal (absolute value). [file 1471-2164-11-192-S2.PDF]

Additional data file 2: Top 50 genes with highest median expression

Feat Num	Gene description	Chr
6573	Unknown	7
980	CLPC (HEAT SHOCK PROTEIN 93-V); ATP binding / ATPase	14
4997	PETC (PHOTOSYNTHETIC ELECTRON TRANSFER C)	1
4749	KOG1441 Glucose-6-phosphate/phosphate antiporter	15
4432	GUN5 (GENOMES UNCOUPLED 5)	4
5045	KOG3283 40S ribosomal protein S8	14
7585	RCA (RUBISCO ACTIVASE)	4
6406	KOG1001 Helicase-like transcription factor HLTF/DNA helicase RAD5	6
4770	KOG3204 60S ribosomal protein L13a	12
2861	60S ribosomal protein L14 (RPL14A)	6
6562	photosystem II protein M	12
1981	ENDOPLASMIC RETICULUM-TYPE CALCIUM-TRANSPORTING ATPASE3	1
6771	ATGCN4 (Arabidopsis thaliana general control non-repressible 4)	15
7363	60S ribosomal protein L18A (RPL18aB)	7
5515	SAPX; L-ascorbate peroxidase	1
3840	31 kDa ribonucleoprotein, chloroplast, putative / RNA-binding protein RNP-T	9
5411	PRK (PHOSPHORIBULOKINASE); ATP binding	4
5007	Ribosomal protein L17 family protein	1
7887	60S ribosomal protein L12 (RPL12C)	3
2649	CXIP1 (CAX INTERACTING PROTEIN 1)	1
3575	ethylene-responsive DEAD box RNA helicase, putative (RH30)	12
7483	Ribosomal protein L13 family protein	3
3536	ARP2/RPL3B (ARABIDOPSIS RIBOSOMAL PROTEIN 2)	1
918	TOM40 (translocase of the outer mitochondrial membrane 40)	3
7177	60S ribosomal protein L11 (RPL11B)	3
5041	MPPBETA; metalloendopeptidase	14
7428	TGD2 (TRIGALACTOSYLDIACYLGLYCEROL2)	6
3572	KOG0764 Mitochondrial FAD carrier protein	1
4780	KOG3254 Mitochondrial/chloroplast ribosomal protein L6	15
1823	KOG1810 Cell cycle-associated protein	6
1552	KOG0675 Calnexin	3
4021	GL3 (GLABRA 3); transcription factor	3
356	ABC transporter family protein	5
5454	CHLI1 (CHLORINA 42); magnesium chelatase	2
408	60S ribosomal protein L7 (RPL7B)	3
2596	cytochrome c oxidase subunit 6b, putative	17
485	40S ribosomal protein S20 (RPS20B)	3
7113	KOG1475 Ribosomal protein RPL1/RPL2/RL4L4	16
4639	KOG0729 26S proteasome regulatory complex, ATPase RPT1	9
7710	Unknown	1
1857	KOG1080 Histone H3 (Lys4) methyltransferase complex, subunit SET1	6
3983	Ribosomal protein L19 family protein	20
7797	KOG0776 Geranylgeranyl pyrophosphate synthase/Polyprenyl synthetase	17
5768	Unknown	17
5488	CLPP5 (NUCLEAR ENCODED CLP PROTEASE 1); endopeptidase Clp	2
897	LPD1 (LIPOAMIDE DEHYDROGENASE 1)	4
315	KOG0465 Mitochondrial elongation factor	7
1663	KOG1711 Mitochondrial/chloroplast ribosomal protein L22	18
267	KOG4153 Fructose 1,6-bisphosphate aldolase	10

Photosynthesis and carbon fixation, ribosomal proteins

Feat Num (Feature Num), Chr (Chromosome number)
