# Supplementary material for: Orchestrated transcription of biological processes in the marine picoeukaryote Ostreococcus exposed to light/dark cycles
Source: BMC Genomics. 2010 Mar 22;11:192. doi: 10.1186/1471-2164-11-192 (PMC2850359; doi:10.1186/1471-2164-11-192)
Supplement: Additional file 3 — Coregulation of genes involved in basic transcription machinery during the night. BFC clusters from 2038 gene probes selected after PCA. Each colour corresponds to a biological process. Feature Number (Feat Num), BFC cluster number (BFC). Stars indicate two probes corresponding to two Feature Numbers associated to a single gene in the final annotation. Bottom right: The main BFC profiles and coefficients are shown. Note that clusters 61, 54, 111 and 67, clusters 29 and 92 as well as clusters 80 and 28 have almost identical profiles and for convenience only one profile (e.g. 61) is shown. [file 1471-2164-11-192-S3.PDF]

Additional data file 3

Transcription and mRNA processing (RNA polymerase, snoRP, RNA splicing, RNA methylase, RNA helicase), Ribosome biogenesis, tRNA and translation

Feat Num	BFC	Gene description
3519	61	40S ribosomal protein S10 (RPS10A)
7616	61	DNA-directed RNA polymerase
3985*	61	KOG0272 U4/U6 small nuclear ribonucleoprotein Prp4 (contains WD40 repeats)
8033*	61	KOG0272 U4/U6 small nuclear ribonucleoprotein Prp4 (contains WD40 repeats)
7455	61	KOG2574 mRNA splicing factor PRP31
6169	61	60S ribosomal protein L10 (RPL10B)
4427	61	KOG0110 RNA-binding protein (RRM superfamily)
3081	61	KOG3503 H/ACA snoRNP complex, subunit NOP10
4169	61	KOG1596 Fibrillarin and related nucleolar RNA-binding proteins
7875	61	KOG2574 mRNA splicing factor PRP31
6369	61	KOG2409 KRR1-interacting protein involved in 40S ribosome biogenesis
6832	61	DEAD box RNA helicase (RH26)
487	61	KOG1253 tRNA methyltransferase
7804	61	DNA binding / DNA-directed RNA polymerase
1770	61	KOG0121 Nuclear cap-binding protein complex, subunit CBP20
5731	61	KOG3073 Protein required for 18S rRNA maturation and 40S ribosome biogenesis
1456	54	KOG0838 RNA Methylase, SpoU family
4501	54	KOG0924 mRNA splicing factor ATP-dependent RNA helicase
3457	54	KOG3064 RNA-binding nuclear protein (MAK16) containing a distinct C4 Zn-finger
423	54	KOG0433 Isoleucyl-tRNA synthetase
5462	54	KOG0603 Ribosomal protein S6 kinase
4689	54	KOG0333 U5 snRNP-like RNA helicase subunit
7951	54	KOG0048 Transcription factor, Myb superfamily
6894	54	KOG2054 Nucleolar RNA-associated protein (NRAP)
5351	54	KOG1801 tRNA-splicing endonuclease positive effector (SEN1)
4745	54	RNA binding
3183	54	KOG4213 RNA-binding protein La
5690	111	DNA-directed RNA polymerase II, putative
6054	111	pseudouridine synthase family protein
7993	111	KOG0302 Ribosome Assembly protein
1475	111	KOG1896 mRNA cleavage and polyadenylation factor II complex, subunit CFT1
4021	67	GL3 (GLABRA 3); transcription factor
6485	67	TRFL6 (TRF-LIKE 6); DNA binding / transcription factor
5403	67	DNA topoisomerase family protein
3045	67	PAB5 (POLY(A)-BINDING PROTEIN); RNA binding
2097	67	SPDS1 (SPERMIDINE SYNTHASE 1)
6271	67	KOG4008 rRNA processing protein RRP7
7209	29	KOG0147 Transcriptional coactivator CAPER (RRM superfamily)
6152	29	KOG3293 Small nuclear ribonucleoprotein (snRNP)
836	29	KOG0466 Translation initiation factor 2, gamma subunit (eIF-2gamma; GTPase)
4540	29	KOG3045 Predicted RNA methylase involved in rRNA processing
5349	29	pseudouridylate synthase
1258	29	KOG2102 Exosomal 3'-5' exoribonuclease complex, subunit Rrp44/Dis3
6259	29	tRNA/rRNA methyltransferase (SpoU) family protein
158	29	nonsense-mediated mRNA decay NMD3 family protein
6253	29	DRH1 (DEAD box RNA helicase 1)
2563	29	KOG2553 Pseudouridylate synthase
1140	29	KOG0343 RNA Helicase
7060	42	KOG2738 Putative methionine aminopeptidase
5502	42	KOG2809 Telomerase elongation inhibitor/RNA maturation protein PINX1
7032	42	KOG1855 Predicted RNA-binding protein
5987	42	KOG3909 Queuine-tRNA ribosyltransferase
7474	42	KOG2793 Putative N2,N2-dimethylguanosine tRNA methyltransferase
874	42	ADK1 (ADENYLATE KINASE 1); adenylate kinase
6533	42	KOG2809 Telomerase elongation inhibitor/RNA maturation protein PINX1
312	42	DEAD box RNA helicase, putative (RH20)
6888	42	RNA cyclase family protein
211	42	DEAD/DEAH box helicase, putative (RH10)
4980	92	APUM23 (ARABIDOPSIS PUMILIO 23); RNA binding
4018	92	nucleolar protein, putative
2930	92	KOG2187 tRNA uracil-5-methyltransferase
1629	92	KOG3492 Ribosome biogenesis protein NIP7
3311	92	KOG1070 rRNA processing protein Rrp5
7136	92	DEAD/DEAH box helicase, putative
5801	92	APUM24 (ARABIDOPSIS PUMILIO 24); RNA binding
5824	92	KOG2038 CAATT-binding transcription factor
1732	92	KOG0272 U4/U6 small nuclear ribonucleoprotein Prp4

6082	80	KOG3115 Methyltransferase-like protein
5955	80	KOG0148 Apoptosis-promoting RNA-binding protein TIA-1/TIAR
486	80	RBM28, rna-binding protein 28 (rna-binding motif protein 28)
2247	80	KOG0343 RNA Helicase
2224	80	KOG1098 Putative SAM-dependent rRNA methyltransferase SPB1
157	28	RNA binding / tRNA (guanine-N2-)-methyltransferase
376	28	dihydrouridine synthase family protein
2911	28	KOG1926 Predicted regulator of rRNA gene transcription (MYB-binding protein)
365	28	KOG1882 Transcriptional regulator SNIP1, contains FHA domain
2070	28	KOG1562 Spermidine synthase
7415	28	40S ribosomal protein S12 (RPS12C)
3297	28	KOG3051 RNA binding/translational regulation protein of the SUA5 family
5306	28	KOG2102 Exosomal 3'-5' exoribonuclease complex, subunit Rrp44/Dis3
7507	12	KOG4661 Hsp27-ERE-TATA-binding protein
3581	12	KOG2529 Pseudouridine synthase
2944	12	KOG1919 RNA pseudouridylate synthases
47	12	KOG0924 mRNA splicing factor ATP-dependent RNA helicase
7680	12	KOG2793 Putative N2,N2-dimethylguanosine tRNA methyltransferase

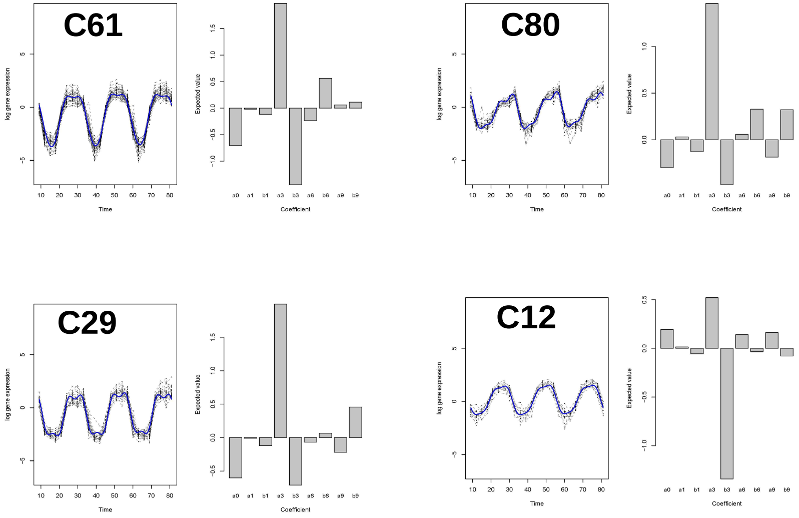

Coregulation of genes involved in basic transcription machinery during the night. BFC clusters from 2038 gene probes selected after PCA. Each colour corresponds to a biological process. Feature Number (Feat Num), BFC cluster number (BFC). Stars indicate two probes corresponding to two Feature Numbers associated to a single gene in the final annotation. Bottom right: The main BFC profiles and coefficients are shown. Note that clusters 61, 54, 111 and 67, clusters 29 and 92 as well as clusters 80 and 28 have almost identical profiles and for convenience only one profile (e.g. 61) is shown.
