# Supplementary material for: Orchestrated transcription of biological processes in the marine picoeukaryote Ostreococcus exposed to light/dark cycles
Source: BMC Genomics. 2010 Mar 22;11:192. doi: 10.1186/1471-2164-11-192 (PMC2850359; doi:10.1186/1471-2164-11-192)
Supplement: Additional file 4 — Clusters of genes involved in protein synthesis including translation regulators, tRNA and amino acid biosynthesis around dawn. BFC clusters from 2038 gene probes selected after PCA. Each colour corresponds to a biological process. Feature Number (Feat Num), BFC cluster number (BFC). Right: The main BFC profiles are shown. Note that clusters 39 and 107 have nearly identical profiles. [file 1471-2164-11-192-S4.PDF]

Additional File 4

Translation factors , amino acid synthesis, tRNA synthesis, ribosome biogenesis and transcription

Feat Num	BFC	Gene description
4992	39	methionine--tRNA ligase, putative / methionyl-tRNA synthetase, putative
4471	39	KOG2240 RNA polymerase II general transcription factor BTF3
8027	39	KOG0628 Aromatic-L-amino-acid/L-histidine decarboxylase
932	39	KOG4492 Chorismate synthase
3437	39	KOG0401 Translation initiation factor 4F,(eIF-4G)
5511	39	translation initiation factor IF-2, chloroplast, putative
6979	39	KOG0433 Isoleucyl-tRNA synthetase
3921	39	translation initiation factor 3 (IF-3) family protein
6395	39	aspartate/glutamate/uridylate kinase family protein
5130	39	KOG2072 Translation initiation factor 3, subunit a (eIF-3a)
2278	39	KOG1801 tRNA-splicing endonuclease positive effector (SEN1)
6326	39	KOG1801 tRNA-splicing endonuclease positive effector (SEN1)
5015	39	KOG0556 Aspartyl-tRNA synthetase
7079	39	KOG1885 Lysyl-tRNA synthetase (class II)
5302	39	KOG3677 RNA polymerase I-associated factor - PAF67
6634	39	KOG1560 Translation initiation factor 3, subunit h (eIF-3h)
4772	39	KOG4655 U3 small nucleolar ribonucleoprotein (snoRNP) component
1197	39	KOG2436 Acetylglutamate kinase/acetylglutamate synthase
6767	39	KOG4163 Prolyl-tRNA synthetase
5791	39	KOG2314 Translation initiation factor 3, subunit b (eIF-3b)
3201	39	60S ribosomal protein L23 (RPL23B)
203	39	SSR16 (ribosomal protein S16); structural constituent of ribosome
474	39	tRNA pseudouridine synthase family protein
1675	107	KOG2975 Translation initiation factor 3, subunit f (eIF-3f)
7489	107	KOG0401 Translation initiation factor 4F, ribosome/ (eIF-4G)
1289	107	KOG2072 Translation initiation factor 3, subunit a (eIF-3a)
5888	107	ribosomal protein L1 family protein
4107	107	KOG3499 60S ribosomal protein L38
6788	107	APG3 (ALBINO AND PALE GREEN); translation release factor
2545	107	APUM12 (ARABIDOPSIS PUMILIO 12); RNA binding
5547	115	KOG0257 Kynurenine aminotransferase, glutamine transaminase K
5963	115	KOG1579 Homocysteine S-methyltransferase
6463	115	KOG4175 Tryptophan synthase alpha chain
5339	115	KOG1637 Threonyl-tRNA synthetase

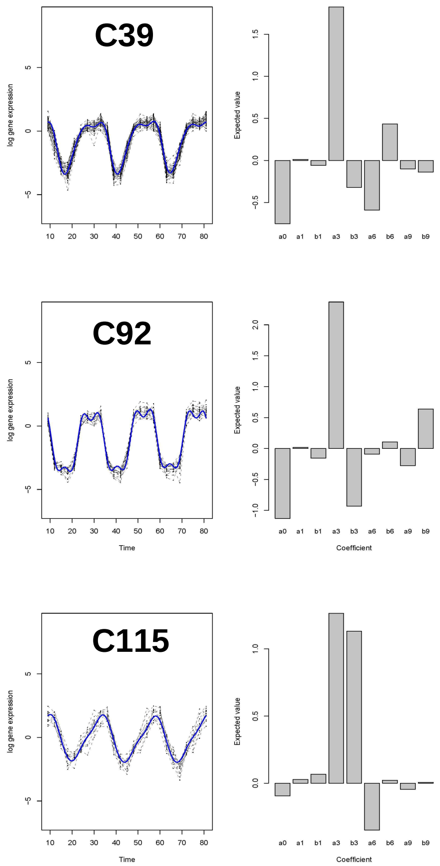

Clusters of genes involved in protein synthesis including translation regulators, tRNA and amino acid biosynthesis around dawn. BFC clusters from 2038 gene probes selected after PCA. Each colour corresponds to a biological process. Feature Number (Feat Num), BFC cluster number (BFC). Right: The main BFC profiles are shown. Note that clusters 39 and 107 have nearly identical profiles.
