# Supplementary material for: Orchestrated transcription of biological processes in the marine picoeukaryote Ostreococcus exposed to light/dark cycles
Source: BMC Genomics. 2010 Mar 22;11:192. doi: 10.1186/1471-2164-11-192 (PMC2850359; doi:10.1186/1471-2164-11-192)
Supplement: Additional file 5 — Coregulation of DNA replication and DNA repair genes at the end of the light period. S Phase BFC Clusters from 2038 gene probes selected after PCA. Each colour corresponds to a biological process. Feature Number (Feat Num), BFC cluster number (BFC). Right: The main BFC profiles and coefficients are shown. Note that clusters 41, 64 and 87 as well as clusters 57 and 116, clusters 46, 122 and 129 have nearly identical profiles. [file 1471-2164-11-192-S5.PDF]

Additional data file 5

DNA replication, DNA repair, chromosome structure, histone and chromatin remodelling , cell cycle control, organelles division, transcription

Feat Num	BFC	Gene description
7024	41	KOG2011 Sister chromatid cohesion complex Cohesin, subunit STAG/IRR1/SCC3
6006	41	KOG0386 Chromatin remodeling complex SWI/SNF, component SWI2)
7990	41	KOG2543 Origin recognition complex, subunit 5
4965	41	KOG1513 Nuclear helicase MOP-3/SNO (DEAD-box superfamily)
5441	41	KOG4603 TBP-1 interacting protein
2614	41	KOG1978 DNA mismatch repair protein - MLH2/PMS1/Pms2 family
5851	41	FTSZ1-1 (FtsZ1-1); structural molecule
2271	41	KOG1625 DNA polymerase alpha-primase complex, polymerase-associated subunit B
3595	41	KOG3108 Single-stranded DNA-binding replication protein A (RPA), medium (30 kD) subunit
6779	41	nucleotide binding
5453	64	KOG3125 Thymidine kinase
3411	64	KOG0481 DNA replication licensing factor, MCM5 component
347	64	KOG3265 Histone chaperone involved in gene silencing
7195	64	PCNA1 (PROLIFERATING CELLULAR NUCLEAR ANTIGEN)
3821	64	ATR (ATAXIA TELANGIECTASIA-MUTATED AND RAD3-RELATED)
5298	64	EMB2411 (EMBRYO DEFECTIVE 2411); ATP-dependent DNA helicase
6286	64	KOG0969 DNA polymerase delta, catalytic subunit
748	64	KOG2299 Ribonuclease HI
7630	64	ARID/BRIGHT DNA-binding domain-containing protein
7526	87	KOG0481 DNA replication licensing factor, MCM5 component
7654	87	KOG2851 Eukaryotic-type DNA primase, catalytic (small) subunit
1998	57	MFP1 (MAR BINDING FILAMENT-LIKE PROTEIN 1)
8049	57	KOG1525 Sister chromatid cohesion complex Cohesin, subunit PDS5
2893	57	ATRAD17 (RADIATION SENSITIVE)
305	57	KOG2807 RNA polymerase II transcription initiation/nucleotide excision repair factor TFIIH
8051	57	KOG0968 DNA polymerase zeta, catalytic subunit
1266	57	MYB3R-5 (myb domain protein 3R-5); DNA binding / transcription factor
2012	116	KOG0996 Structural maintenance of chromosome protein 4 (Condensin, subunit C)
4534	116	WEE1 (Arabidopsis wee1 kinase homolog); kinase/ protein kinase
1785	116	KOG3786 RNA polymerase II assessory factor Cdc73p
3244	116	KOG4373 Predicted 3'-5' exonuclease
6438	116	ERCC1 (UV REPAIR DEFICIENT 7)
5055	46	KOG1112 Ribonucleotide reductase, alpha subunit
4903	46	ARC5 (ACCUMULATION AND REPLICATION OF CHLOROPLAST 5); GTP binding / GTPase
5585	46	KOG1744 Histone H2B
3730	46	CDKB (CDC2-LIKE GENE) kinase
4507	122	histone H3.2
1916	122	MFP1 (MAR BINDING FILAMENT-LIKE PROTEIN 1)
7336	122	kinesin motor protein-related
3193	129	KOG0642 Cell-cycle nuclear protein, contains WD-40 repeats
7574	129	KOG0220 Mismatch repair ATPase MSH4 (MutS family)
2589	129	KOG4109 Histone H3 (Lys4) methyltransferase complex, subunit CPS25/DPY-30
3608	129	KOG0851 Single-stranded DNA-binding replication protein A (RPA), large (70 kD)
3410	109	ARC6 (ACCUMULATION AND REPLICATION OF CHLOROPLASTS 6)
2185	109	KOG0386 Chromatin remodeling complex SWI/SNF, component SWI2 (DNA/RNA helicase)
6362	109	KOG2519 5'-3' exonuclease
6318	109	KOG0481 DNA replication licensing factor, MCM5 component
1466	109	KOG2097 Predicted N6-adenine methylase involved in transcription regulation
1228	102	cell division cycle protein 48-related / CDC48-related
3242	102	KOG1513 Nuclear helicase MOP-3/SNO (DEAD-box superfamily)

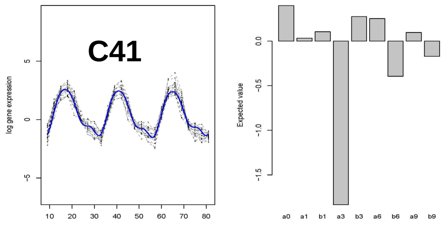

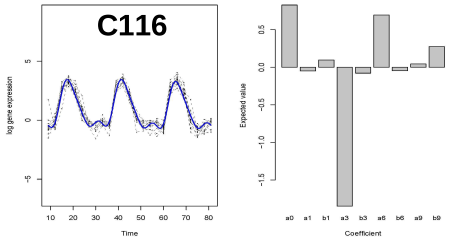

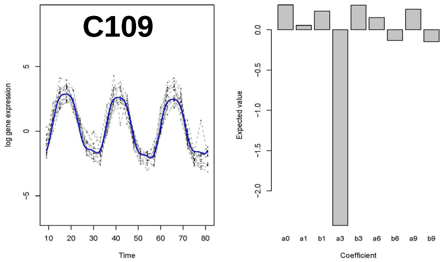

Coregulation of DNA replication and DNA repair genes at the end of the light period. S Phase BFC Clusters from 2038 gene probes selected after PCA. Each colour corresponds to a biological process. Feature Number (Feat Num), BFC cluster number (BFC). Right: The main BFC profiles and coefficients are shown. Note that clusters 41, 64 and 87 as well as clusters 57 and 116, clusters 46, 122 and 129 have nearly identical profiles.
