# Supplementary material for: Orchestrated transcription of biological processes in the marine picoeukaryote Ostreococcus exposed to light/dark cycles
Source: BMC Genomics. 2010 Mar 22;11:192. doi: 10.1186/1471-2164-11-192 (PMC2850359; doi:10.1186/1471-2164-11-192)
Supplement: Additional file 6 — Cluster of genes involved in photoprotection, defence against oxidative stress and DNA repair around midday. BFC clusters from 2038 gene probes selected after PCA. Each colour corresponds to a biological process. Feature Number (Feat Num), BFC cluster number (BFC). Right: The main BFC profiles and coefficients are shown. [file 1471-2164-11-192-S6.PDF]

Additional data file 6

Oxidative stress, carotenoids biosynthesis, DNA repair and remodelling, photosynthesis and lipid metabolism

Feat Num	BFC	Gene description
2303	85	thioredoxin-related
4249	85	ACCELERATED CELL DEATH 1, PHEOPHORBIDE A OXYGENASE
5831	85	ATF1/TRXF1 (THIOREDOXIN F-TYPE 1)
2506	85	KOG0191 Thioredoxin/protein disulfide isomerase
3791	85	thylakoid lumen 18.3 kDa protein
2186	85	NPQ1 (NON-PHOTOCHEMICAL QUENCHING 1)
3897	27	UVR3 (UV REPAIR DEFECTIVE 4)
6562	27	photosystem II protein M
2550	27	ATMINE1 (ARABIDOPSIS HOMOLOGUE OF BACTERIAL MINE 1)
143	27	CSD2 (COPPER/ZINC SUPEROXIDE DISMUTASE 2)
7017	7	FORMAMIDOPYRIMIDINE-DNA GLYCOSYLASE 1,
807	7	DNA cross-link repair protein-related
2184	7	CHR17 (CHROMATIN REMODELING FACTOR17); DNA-dependent ATPase
2538	7	DEAD/DEAH box helicase, putative (RH22)
3014	7	ATR2 (ARABIDOPSIS P450 REDUCTASE 2)
7850	7	violaxanthin de-epoxidase-related
7938	7	GPPS (GERANYLPYROPHOSPHATE SYNTHASE)
6921	7	ATERS/ERS/OVA3 (OVULE ABORTION 3); glutamate-tRNA ligase
3607	7	PGR5 (PROTON GRADIENT REGULATION 5)
7870	24	KOG4720 Ethanolamine kinase
7800	24	KOG4254 Phytoene desaturase
6238	24	ABA1 (ABA DEFICIENT 1); zeaxanthin epoxidase
4791	24	HEMA1; glutamyl-tRNA reductase
4877	24	KOG1336 Monodehydroascorbate/ferredoxin reductase
7861	24	KOG4232 Delta 6-fatty acid desaturase/delta-8 sphingolipid desaturase
7480	76	FAD2 (FATTY ACID DESATURASE 2); delta12-fatty acid dehydrogenase
6483	76	KOG1737 Oxysterol-binding protein
6947	76	KOG1285 Beta, beta-carotene 15,15'-dioxygenase
4703	76	CLB6 (CHLOROPLAST BIOGENESIS 6)
1285	76	PDE149 (PIGMENT DEFECTIVE 149)
5231	76	FAD2 (FATTY ACID DESATURASE 2); delta12-fatty acid dehydrogenase

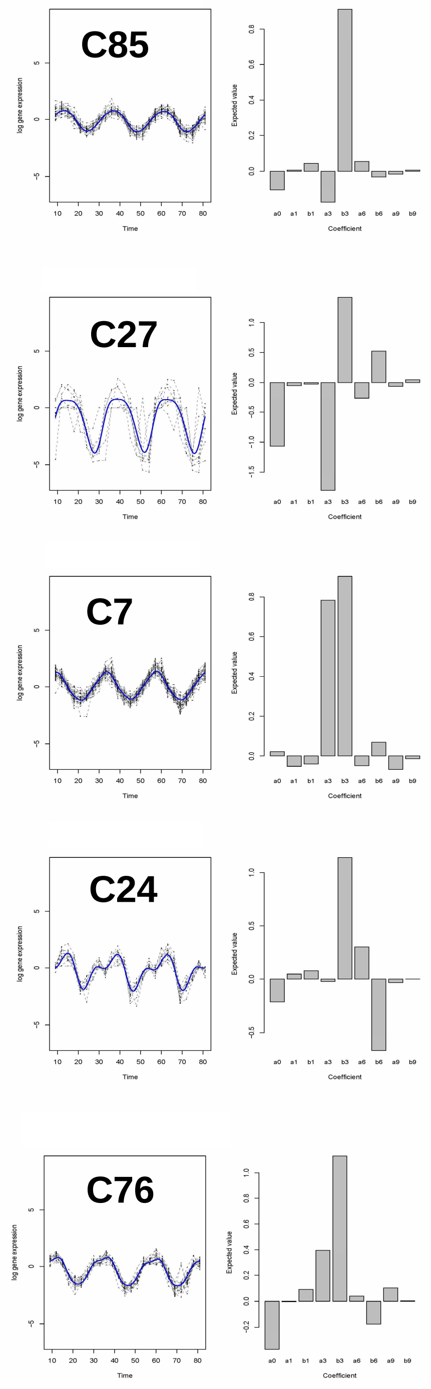

Cluster of genes involved in photoprotection, defence against oxidative stress and DNA repair around midday. BFC clusters from 2038 gene probes selected after PCA. Each colour corresponds to a biological process. Feature Number (Feat Num), BFC cluster number (BFC). Right: The main BFC profiles and coefficients are shown.
