# Supplementary material for: Orchestrated transcription of biological processes in the marine picoeukaryote Ostreococcus exposed to light/dark cycles
Source: BMC Genomics. 2010 Mar 22;11:192. doi: 10.1186/1471-2164-11-192 (PMC2850359; doi:10.1186/1471-2164-11-192)
Supplement: Additional file 7 — Coregulation of genes involved in mitosis at dusk. Mitotic BFC clusters from 2038 gene probes selected after PCA. Each colour corresponds to a biological process. Feature Number (Feat Num), BFC cluster number (BFC). Right: The main BFC profiles and coefficients are shown. Note that clusters 71 and 49 as well as clusters 104 and 36 have nearly identical profiles. [file 1471-2164-11-192-S7.PDF]

Additional data file 7

Cytoskeleton, regulatory kinases, DNA replication and repair, chromosome structure, secretion, oxidative stress and iron

Feat Num	BFC	Gene description
6489	71	KOG2328 Chromosome condensation complex Condensin, subunit H
4976	71	KOG0414 Chromosome condensation complex Condensin, subunit D2
7432	71	ATORC1B/ORC1B/UNE13 (ORIGIN OF REPLICATION COMPLEX 1B)
4436	71	CHR24 (chromatin remodeling 24); ATP binding / DNA binding / helicase
388	71	KOG1068 Exosomal 3'-5' exoribonuclease complex, subunit Rrp41 and related exoribonucleases
3924	71	ATK2 (ARABIDOPSIS THALIANA KINESIN 2); microtubule motor
5956	71	KOG0517 Beta-spectrin
7691	71	KOG2464 Serine/threonine kinase (haspin family)
788	71	POK1 (PHRAGMOPLAST ORIENTING KINESIN 1); microtubule motor
790	71	KOG1433 DNA repair protein RAD51/RHP55
4287	71	ATK5 (Arabidopsis thaliana kinesin 5); microtubule motor
4259	71	KOG0192 Tyrosine kinase specific for activated (GTP-bound) p21cdc42Hs
4784	71	ATEB1C (MICROTUBULE END BINDING PROTEIN 1); microtubule binding
6717	71	KOG2084 Predicted histone tail methylase containing SET domain
1967	49	CYCB2;2 (CYCLIN B2;2); cyclin-dependent protein kinase regulator
1146	49	KOG0161 Myosin class II heavy chain
7911	49	KOG2058 Ypt/Rab GTPase activating protein
2707	49	KOG2025 Chromosome condensation complex Condensin, subunit G
6733	104	KOG2510 SWI-SNF chromatin-remodeling complex protein
5593	104	KOG0161 Myosin class II heavy chain
1088	104	CKS1 (CDK-SUBUNIT 1); cyclin-dependent protein kinase
5330	104	ATAUR1 (ATAURORA1); histone serine kinase(H3-S10 specific) / kinase/ protein serine/threonine kinase
7755	104	KOG0192 Tyrosine kinase specific for activated (GTP-bound) p21cdc42Hs
7463	36	KOG0239 Kinesin (KAR3 subfamily)
3385	36	SMC2 (STRUCTURAL MAINTENANCE OF CHROMOSOMES 2)
8020	77	KOG1651 Glutathione peroxidase
5409	77	KOG2065 Gamma-tubulin ring complex protein
5342	77	KOG0916 1,3-beta-glucan synthase/callose synthase catalytic subunit
3275	77	KOG0605 NDR and related serine/threonine kinases
4264	77	KOG0819 Annexin
5069	77	MOR1 (MICROTUBULE ORGANIZATION 1)
2312	62	KOG0239 Kinesin (KAR3 subfamily)
7847	62	APX1 (ASCORBATE PEROXIDASE 1, MATERNAL EFFECT EMBRYO ARREST 6)
2795	62	KOG1003 Actin filament-coating protein tropomyosin
1181	25	KOG1010 Rb (Retinoblastoma tumor suppressor)-related protein
2326	25	ferredoxin family protein
2886	25	KOG0578 p21-activated serine/threonine protein kinase
4001	25	LEM3 (ligand-effect modulator 3) family protein / CDC50 family protein
5633	25	ATFER3 (FERRITIN 3); ferric iron binding
1318	25	MSH6 (MUTS HOMOLOG 6-1)

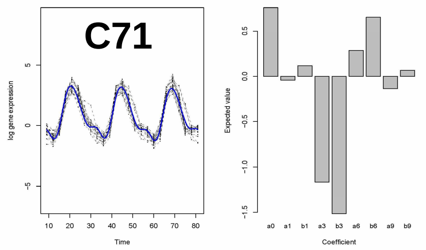

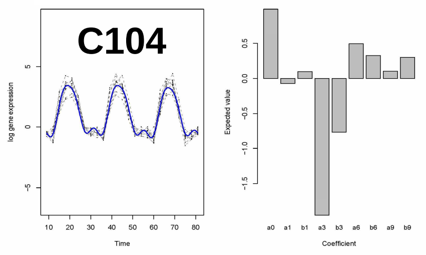

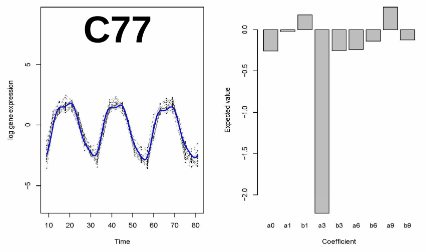

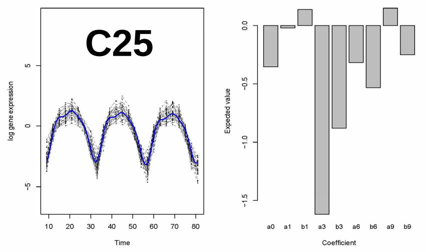

Coregulation of genes involved in mitosis at dusk. Mitotic BFC clusters from 2038 gene probes selected after PCA. Each colour corresponds to a biological process. Feature Number (Feat Num), BFC cluster number (BFC). Right: The main BFC profiles and coefficients are shown. Note that clusters 71 and 49 as well as clusters 104 and 36 have nearly identical profiles.
