# Supplementary material for: Orchestrated transcription of biological processes in the marine picoeukaryote Ostreococcus exposed to light/dark cycles
Source: BMC Genomics. 2010 Mar 22;11:192. doi: 10.1186/1471-2164-11-192 (PMC2850359; doi:10.1186/1471-2164-11-192)
Supplement: Additional file 8 — Late night clusters of genes involved in chloroplast biogenesis, pigment biosynthesis, lipid biosynthesis and metabolism. BFC clusters from 2038 gene probes selected after PCA. Each colour corresponds to a biological process. Feature Number (Feat Num), BFC cluster number (BFC). Right: The main BFC profiles and coefficients are shown. Note that clusters 33 and 6 have nearly identical profiles. [file 1471-2164-11-192-S8.PDF]

Additional data file 8

Chloroplast biogenesis and chlorophyll biosynthesis/degradation, lipid metabolism carotenoids biosynthesis, metabolism

Feat Num	BFC	Gene description
6521	33	3-oxoacyl-(acyl-carrier protein) reductase, chloroplast
4009	33	CHLI2; magnesium chelatase
4579	33	geranylgeranyl reductase
5495	33	KAS I (3-KETOACYL-ACYL CARRIER PROTEIN SYNTHASE I)
3943	33	KOG4232 Delta 6-fatty acid desaturase/delta-8 sphingolipid desaturase
5411	33	PRK (PHOSPHORIBULOKINASE)
897	33	LPD1 (LIPOAMIDE DEHYDROGENASE 1)
1506	33	fructose-bisphosphate aldolase, putative
5664	33	GC1 (GIANT CHLOROPLAST 1); catalytic/ coenzyme binding
6389	33	CH1 (CHLORINA 1); chlorophyll a oxygenase
4564	33	cytochrome b6
5066	33	TIM (TRIOSEPHOSPHATE ISOMERASE)
2820	33	GcpE (CHLOROPLAST BIOGENESIS 4)
3625	33	KOG3773 Adiponutrin and related vesicular transport proteins
1001	33	ACD1 (ACCELERATED CELL DEATH 1, PHEOPHORBIDE A OXYGENASE)
1021	33	GGPS1 (GERANYLGERANYL PYROPHOSPHATE SYNTHASE 1)
6294	33	APE1 (ACCLIMATION OF PHOTOSYNTHESIS TO ENVIRONMENT)
6278	33	FAB1 (FATTY ACID BIOSYNTHESIS 1); fatty-acid synthase
202	33	ATSS3 (STARCH SYNTHASE 3); starch synthase/ transferase
3207	33	KOG1448 Ribose-phosphate pyrophosphokinase
182	33	KOG1470 Phosphatidylinositol transfer protein PDR16 and related proteins
875	6	KOG1458 Fructose-1,6-bisphosphatase
7194	6	RPE (EMBRYO DEFECTIVE 2728); ribulose-phosphate 3-epimerase
1808	6	LUT1 (LUTEIN DEFICIENT 1); oxygen binding
267	6	KOG4153 Fructose 1,6-bisphosphate aldolase
3775	6	PDS3 (PHYTOENE DESATURASE)
5757	6	3,8-divinyl protochlorophyllide a 8-vinyl reductase

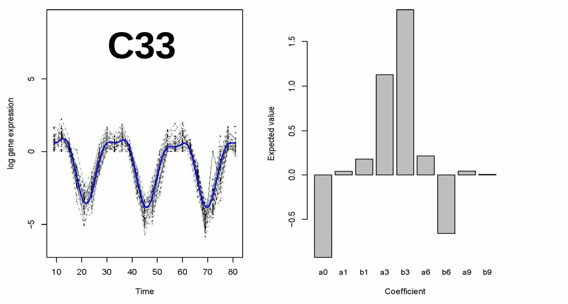

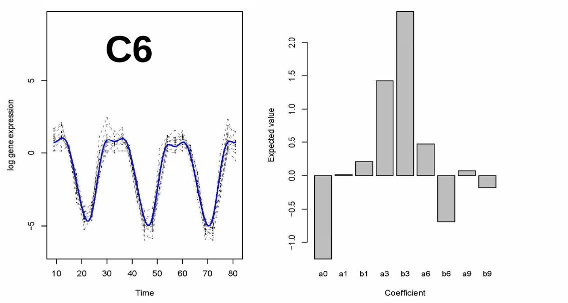

Late night clusters of genes involved in chloroplast biogenesis, pigment biosynthesis, lipid biosynthesis and metabolism. BFC clusters from 2038 gene probes selected after PCA. Each colour corresponds to a biological process. Feature Number (Feat Num), BFC cluster number (BFC). Right: The main BFC profiles and coefficients are shown. Note that clusters 33 and 6 have nearly identical profiles.
