# Supplementary material for: Orchestrated transcription of biological processes in the marine picoeukaryote Ostreococcus exposed to light/dark cycles
Source: BMC Genomics. 2010 Mar 22;11:192. doi: 10.1186/1471-2164-11-192 (PMC2850359; doi:10.1186/1471-2164-11-192)
Supplement: Additional file 9 — Afternoon genes involved in Chlorophyll and Photosystem proteins biosynthesis. BFC clusters from 2038 gene probes selected after PCA. Each colour corresponds to a biological process. Feature Number (Feat Num), BFC cluster number (BFC). Stars indicate two probes corresponding to two Feature Numbers associated to a single gene in the final annotation. Right: The main BFC profiles and coefficients are shown. [file 1471-2164-11-192-S9.PDF]

Additional data file 9

Chlorophyll and photosystem biosynthesis, lipid metabolism

Feat Num	BFC	Gene description
450	26	KOG2872 Uroporphyrinogen decarboxylase
6703	26	HEMB1; porphobilinogen synthase
7529	26	HEMB1; porphobilinogen synthase
6976	26	LIN2 (LESION INITIATION 2); coproporphyrinogen oxidase
6175*	26	lil3 protein, putative
1865*	26	CHLD/PDE166 (PIGMENT DEFECTIVE 166); magnesium chelatase
5212	26	PSBO-2/PSBO2 (PHOTOSYSTEM II SUBUNIT O-2); oxygen evolving
5099	26	GSA2 (GLUTAMATE-1-SEMIALDEHYDE 2,1-AMINOMUTASE 2)
1047	26	GSA2 (GLUTAMATE-1-SEMIALDEHYDE 2,1-AMINOMUTASE 2)
4368	13	PORA (Protochlorophyllide reductase A)
6791	13	CH1 (CHLORINA 1); chlorophyll a oxygenase
67	13	HEME1; uroporphyrinogen decarboxylase
2951	112	LHCA2 (Photosystem I light harvesting complex gene 2)
4633	112	LHCA2 (Photosystem I light harvesting complex gene 2)
4297	112	PSBY (photosystem II BY)
2720	112	PSBP-2 (photosystem II subunit P-2); calcium ion binding
4520	31	LHCA1
691	31	PSAD-2 (photosystem I subunit D-2)
166	31	LHCB4.3 (LIGHT HARVESTING COMPLEX PSII); chlorophyll binding
3323	9	FLU (FLUORESCENT IN BLUE LIGHT); binding
4040	9	LPA1 (LOW PSII ACCUMULATION1); binding
1049	9	thylakoid lumen 15.0 kDa protein
1440	88	photosystem II reaction center W (PsbW) protein-related
4983	88	plastid-lipid associated protein PAP / fibrillin family protein
7663	88	photosystem II family protein
6306	88	CIP1 (COP1-INTERACTIVE PROTEIN 1)
1270	88	family II extracellular lipase, putative

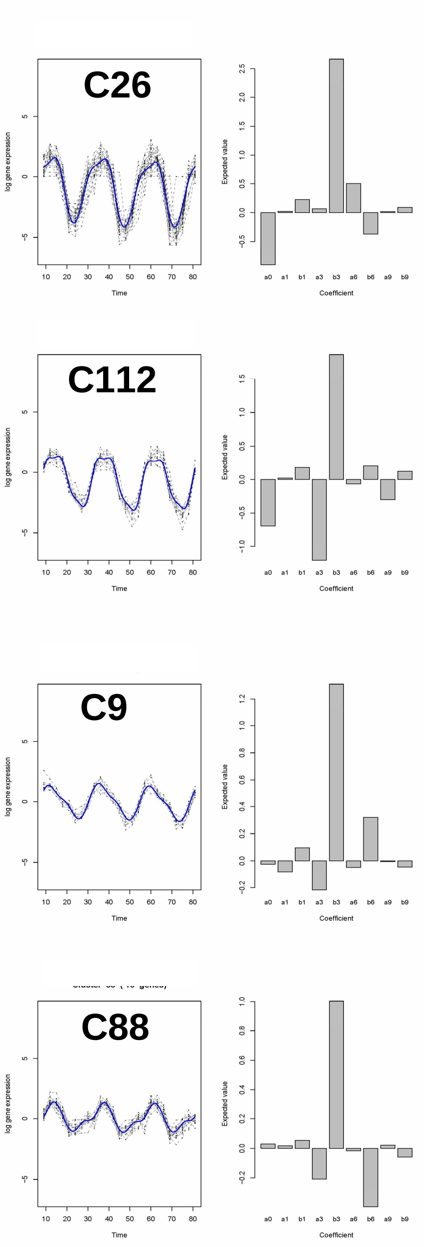

Afternoon genes involved in Chlorophyll and Photosystem proteins biosynthesis. BFC clusters from 2038 gene probes selected after PCA**.** Each colour corresponds to a biological process. Feature Number (Feat Num), BFC cluster number (BFC). Stars indicate two probes corresponding to two Feature Numbers associated to a single gene in the final annotation. Right: The main BFC profiles and coefficients are shown. Note that clusters 26 and 13 as well as clusters 112 and 31 have nearly identical profiles.
