# Supplementary material for: Orchestrated transcription of biological processes in the marine picoeukaryote Ostreococcus exposed to light/dark cycles
Source: BMC Genomics. 2010 Mar 22;11:192. doi: 10.1186/1471-2164-11-192 (PMC2850359; doi:10.1186/1471-2164-11-192)
Supplement: Additional file 10 — Transcriptional regulations of transcription factors. (A) Hierarchical clustering of 170 expressed transcription factors. (B) Transcription patterns of expression of CCAAT HAP3/HAP5 transcription factors. [file 1471-2164-11-192-S10.PDF]

# Additional data file 10

## Transcription factors (170)

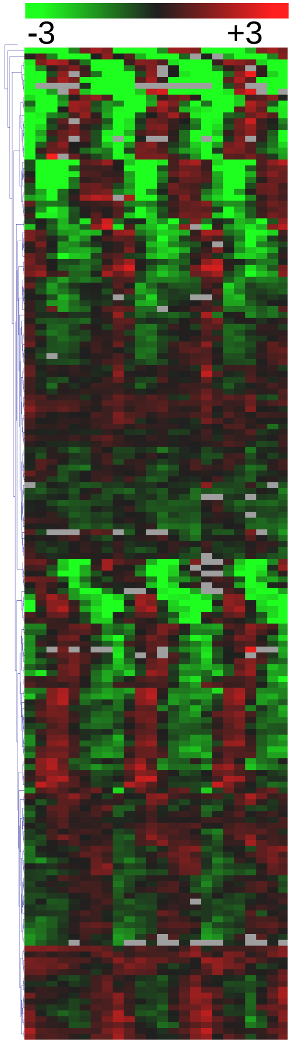


## CCAAT HAP3/HAP5

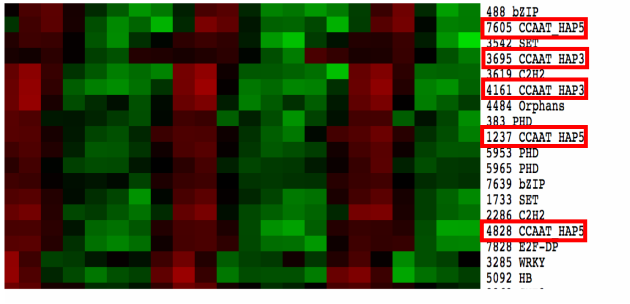


**Transcriptional regulations of transcription factors. (A)** Hierarchical clustering of 170 expressed transcription factors. **(B)** Transcription patterns of expression of CCAAT HAP3/HAP5 transcription factors.
